# Immune and hematologicak responses to the third dose of an mRNA COVID-19 vaccine: a six-month longitudinal study

**DOI:** 10.3389/fcimb.2025.1615227

**Published:** 2025-07-10

**Authors:** Waleed M. Bawazir, Ahmad Al Ibad, Muneeba Mohsin, Hanouf A. Niyazi, Turki A. Alamri, Mohammed A. Bazuhair, Mohannad Hazzazi, Noura A. Chehab, Steve Harakeh, Yasar Mehmood Yousafzai

**Affiliations:** ^1^ Department of Medical Laboratory Sciences, Faculty of Applied Medical Sciences, King Abdulaziz University, Jeddah, Saudi Arabia; ^2^ Hematology Research Unit, King Fahd Medical Research Center, King Abdulaziz University, Jeddah, Saudi Arabia; ^3^ Institute of Pathology and Diagnostic Medicine, Khyber Medical University, Peshawar, Pakistan; ^4^ Department of Pathology, Bannu Medical College, Bannu, Pakistan; ^5^ Department of Biochemistry, Institute of Chemical and Life Sciences, Abdul Wali Khan University, Mardan, Pakistan; ^6^ Department of Clinical Microbiology and Immunology, Faculty of Medicine, King Abdulaziz University, Jeddah, Saudi Arabia; ^7^ Family and Community Medicine Department, Faculty of Medicine in Rabigh, King Abdulaziz University, Jeddah, Saudi Arabia; ^8^ Center of Excellence for Drug Research and Pharmaceutical Industries, King Abdulaziz University, Jeddah, Saudi Arabia; ^9^ Department of Clinical Pharmacology, Faculty of Medicine, King Abdulaziz University, Jeddah, Saudi Arabia; ^10^ NEOM Energy & Water Company (ENOWA), Neom, Saudi Arabia; ^11^ EcoHealth Unit, 14 King Fahd Medical Research Center, King Abdulaziz University, Jeddah, Saudi Arabia; ^12^ Yousef Abdul Latif Jameel Scientific Chair of Prophetic Medicine Application, Faculty of Medicine (FM), King Abdulaziz University, Jeddah, Saudi Arabia; ^13^ Department of Pathology, Rehman Medical Institute, Peshawar, Pakistan

**Keywords:** AEFIs, coagulation profile, complete blood counts, IgA, IgG, inflammatory cytokines, COVID-19 mRNA vaccine

## Abstract

The deployment of mRNA vaccines against SARS-CoV-2 is a major landmark in controlling the COVID-19 pandemic. However, the activation of adaptive immunity and its longevity after a booster dose warrant further investigation. Moreover, the interplay between inflammation and immune thrombosis after transfection needs further insights that could help examine the vaccine’s potential for adverse events following immunization (AEFIs). This study investigates the biochemical and hematological responses to the third dose of a COVID-19 mRNA vaccine in 68 healthy participants who had previously received two doses of the vaccine. Blood samples were collected at baseline (before vaccine dose; D0), 48 hours post-vaccination (D2), and then at days 30, 60, 120, and 180 (D30, D60, D120, D180). The study focused on analyzing changes in anti-SARS-COV-2 immunoglobulins (IgG and IgA), inflammatory biomarkers (IL-6, IFN-γ, CRP, hs-CRP), coagulation factors (PT, APTT, D-dimers), and blood cell counts (neutrophils, leukocytes, platelets) at D2 post-vaccination, and IgG and IgA at days 2, 30, 60, 120, and 180 post-vaccination. In this study, no clinical AEFIs were observed in any of the recipients. Slight changes were observed in the levels of inflammatory and coagulation biomarkers, and blood cells. Levels of CRP and hs-CRP increased slightly but significantly, d-dimers were raised, and PT and aPTT were prolonged significantly. A small but significant decrease was observed in IFN-γ and mean lymphocyte counts, whereas no change was observed in the levels of IL-6, neutrophils, and platelet count at D2. Levels of IgG and IgA showed sustained increase over the six-month period. These results collectively demonstrate that the third dose of the mRNA vaccine elicits a rapid and sustained immune response characterized by increased IgG and IgA levels. The changes observed in inflammatory markers and coagulation factors after vaccination observed shortly after vaccination require further investigations.

## 1 Introduction

The rapid development of mRNA vaccines, such as the Pfizer-BioNTech BNT162b2 vaccine, against SARS-CoV-2, the virus responsible for the COVID-19 pandemic, has been instrumental in controlling disease transmission, morbidity, and mortality (Polack et al., 2020). These vaccines have been shown to induce innate and adaptive immune responses rapidly. These responses include the production of neutralizing antibodies and activating antigen-specific CD4 and CD8 T cells (Gebre et al., 2021; Cagigi and Loré, 2021). The innate immune response is enhanced following booster immunization, leading to a more potent immune response (Arunachalam et al., 2021). The Pfizer-BioNTech vaccine, in particular, has demonstrated high efficacy in preventing symptomatic COVID-19 and reducing the risk of severe disease, hospitalization, and death (Polack et al., 2020).

The immune response comes at the cost of changes in inflammatory and coagulation profiles. These changes have been implied to affect the reported adverse events following immunization. Commonly reported adverse events include pain, redness, swelling at the injection site, fatigue, headache, muscle pain, chills, fever, and nausea. These are generally short-lived, not serious, and indicate the body’s immune response to the vaccine. However, serious adverse events, though rare, have been observed, including allergic reactions, myocarditis, and pericarditis, particularly in young men after the second dose (Yasmin et al., 2023; Seyedalinaghi et al., 2022; Klein et al., 2021). Thromboembolic events and Guillain-Barré syndrome have also been reported, but their association with the vaccine is well elucidated. Although the biological mechanisms underlying the adverse events have not been fully understood, they are postulated to be closely related to inflammatory profile and coagulation system changes that go beyond their normal, necessary threshold.

This study aims to investigate the nuanced biochemical and hematological responses following the third dose of a COVID-19 mRNA vaccine, with a particular focus on the longitudinal immune response. By investigating the dynamics of immunoglobulins (IgG and IgA), inflammatory biomarkers (IL-6, IFN-γ, CRP, hs-CRP), coagulation factors (PT, APTT, D-dimers), and hematological parameters (neutrophils, leukocytes, platelets), we aim to uncover novel insights into the complex mechanisms underlying vaccine-induced immunity and make a way for further research to demonstrate the interplay between these closely linked systems. This research seeks to bridge critical knowledge gaps regarding the long-term effects of booster vaccination, ultimately informing evidence-based strategies for optimizing vaccine efficacy and safety, particularly in high-risk populations.

## 2 Materials and methods

### 2.1 Study participants

This study was initiated after approval from the ethical committee of Khyber Medical University, Peshawar (Letter no: DIR/KMU-EB/KC/000987/DR). It was an observational longitudinal study conducted at Khyber Medical University, Peshawar, from 15 April to 15 November 2022. A non-probability simple, convenient sampling method was used to enroll participants that consisted of 68 healthy individuals aged 20–30 years who had been vaccinated with two doses of a vaccine –the mix of Cansino and Sinovac – at least six months ago and at most eight months ago were included in the study. Participants with COVID-19 infection in less than six months were not included as Government recommended to not vaccinate unless more than six months have passed after the COVID-19 infection. Individuals with a severe allergic reaction to any of the previous doses, profound comorbidities e.g. morbid obesity, diabetes hypertension, ongoing COVID-19 infection and any other infection, any physiological stress e.g. pregnancy, puerperium, immunodeficiency e.g. AIDS, immunosuppressant drugs and any type of bleeding diathesis were excluded. The formula (n=z2x p(1−P)ⅇ2) was used to calculate the sample size where z (confidence level) was 75%, e (margin of error) was 7% and p (estimated proportion of population) was 50%.

Standard ethical principles were followed. The participants’ informed consents were duly signed before the data and blood samples were collected, and they were free to leave the study at any time throughout the follow-up. Clinical advice and assistance were provided to the participants in cases of adverse reactions following vaccination.

### 2.2 Sample collection and processing

Five ml blood was collected before the administration of the Pfizer/BioNTech mRNA vaccine (Batch/Lot No# 36310BA; expiry date: 08/2022; Pfizer Inc. New York, NY 10017, BioNTech Manufacturing GmbH, An der Goldgrube 1255131 Mainz, Germany) & 48 hours after vaccine through vacutainer. Blood was collected in EDTA tubes, Tri-sodium citrate tubes, & Gel tubes. CBC was done from the blood in EDTA tubes using three parts hematology analyzer called Sysmex XP-100 [using cell pack (AM2014/17-09-023) and stromatolyser (AM1026/15-11-022)] & then discarded. Blood collected in Tri-sodium citrate tubes & Gel tubes was centrifuged at 4,500 rpm for 5 min to extract plasma & serum, respectively. Plasma & serum separation were transferred to serum cups & preserved at 4–5 °C & -80 °C, respectively. They were thawed according to the standard protocol before analysis. Plasma was analyzed for PT & APTT through manual method. Using Finecare FIA FS-112, serum was analyzed for CRP and hs-CRP (test kit Ref#W201, LOT#F20115C0EAD) and D-dimers (Ref#211, LOT#F2111560DAD). Serum was also analyzed for IL-6 & IFN-γ using BioTek ELISA machine (EL x800) and kits from BT LAB (Catalogue. No. E0090Hu, LOT. No. 202210011; Bioassay Technology Laboratory, Birmingham, England) & (Catalogue. No. E0105Hu, LOT. No. 202210011; Bioassay Technology Laboratory, Birmingham, England). Samples after that, i.e., from day 30 to day 180, were collected only in Gel tubes & centrifuged at 4,500 rpm for 5 minutes. Serum separated was collected in serum cups & preserved at -80 °C. Serum from all samples was analyzed for Anti-SARS CoV2 Anti-RBD IgG & IgA through BioTek ELISA machine (EL x800) using ELISA kit from FineTest (Catalogue. No. EH4943, Batch. No. H4850H103; Wuhan Fine Biotech Co., LTD, Optics Valley Biomedical Industrial Park, Wuhan, China) and (Catalogue. No. EH4950, Batch. No. H4950H106 J; Wuhan Fine Biotech Co., LTD, Optics Valley Biomedical Industrial Park, Wuhan, China), respectively.

### 2.3 Florescence immunoassay

CRP, hs-CRP & D-dimers were analyzed using Finecare FIA FS-112 machine, which uses a sandwich immune detection method. 5-µl serum for CRP & hs-CRP & 10-µl plasma for D-dimers were transferred from serum cups to the detection tube with a transfer pipette. The detection buffer lid was closed and shaken gently ten times to mix the sample thoroughly. 75 µl of sample mixture was taken and loaded into the sample well of the test cartridge, which was then inserted into the cartridge holder of the FIA machine & then run. Results were displayed on the main screen of the machine system.

### 2.4 Indirect enzyme-linked immunoassay

Quantitative anti-SARS CoV-2 IgG and anti-SARS CoV-2 IgA antibody kits performed ELISA following the manufacturer’s guidelines. Reagents were brought to room temperature. Plates were washed two times before adding standard, sample (diluted 1/100 with sample solution buffer) & control (Blank) well. 50µl standard or sample was added to each well & incubated for 30 minutes at 37 °C. Plates were aspirated & washed three times. 50µl HRP-labeled antibody working solution was added to each well & incubated for 30 minutes at 37°C. Plates were again aspirated & washed five times. 50µl TMB substrate solution was added & incubated for 15 minutes at 37 °C. 50µl stop solution was added, then the plates were inserted into the plate reader & read at 450 nm immediately.

Quantitative IFN-γ and IL-6 kits performed ELISA following the manufacturer’s guidelines. Reagents were brought to room temperature. Plates were washed two times before adding standard, sample (diluted 1/20 with sample solution buffer) & control (Blank) well. 50µl standard was added to standard wells & 40µl sample to sample wells. 10µl anti-IFN-γ and anti-IL-6 antibodies were added to sample wells, followed by 50µl streptavidin-HRP to both sample and standard wells. The plate was covered with sealer & incubated for 60 minutes at 37°C. Plates were washed five times with wash buffer & 50 µl substrate solution A was added to each well, followed by 50 µl substrate solution B. Plates were then sealed and incubated for 10 minutes at C. 50 µl of stop solution was added to all wells, changing blue to yellow. Plates were inserted into the p37 °C late reader within 10 minutes and read at 450 nm.

### 2.5 Statistical analysis

The study data were exported to an MS Excel sheet and analyzed using GraphPad Prism (Version: 9.5.1, Company: Dotmatics, Country: United States). The mean and standard deviation were computed for continuous numerical variables, while frequency and percentages were calculated for discrete categorical variables. A P-value was obtained to determine the statistical significance of the tests.

## 3 Results

In this study, 68 individuals were enrolled. Their ages ranged between 20 and 30. Among the 68 participants, 41% (n=28) were male, while 59% (n=40) were female. No differences were made based on age or gender. Moreover, 28% (n=19) reported that they had not contracted symptomatic infection, while 72% (n=49) reported that they had suffered from symptomatic infection before the third dose of vaccine, which PCR confirmed.

### 3.1 Anti-SARS-CoV-2 antibody levels

Anti-SARS-CoV-2 IgG and IgA levels were assessed at six-time points through ELISA at day 0 (immediately before the vaccine’s administration) and then at days 2, 30, 60, 120, and 180 post-vaccine administration.

**Figure 1:**
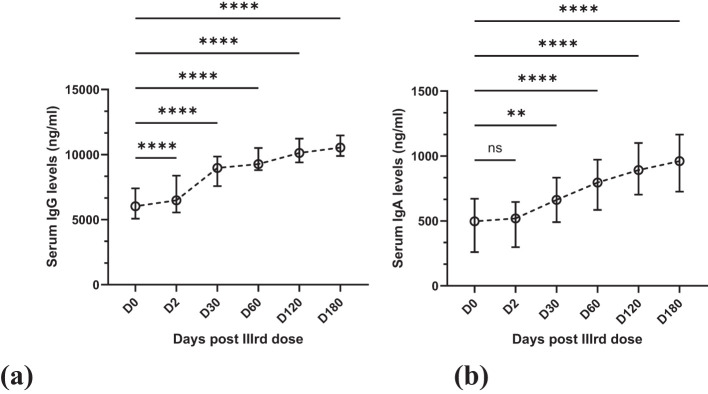
Line graphs showing the gradual increase in antibody levels over six months. Statistical test: Kruskal-Wallis with *post-hoc* Dunn’s test for pairwise multiple comparisons. **(a)**. Mean IgG with SD in serum over six months at six-time points. **(b)** Mean IgA with SD in serum over six months at six-time points. ns (not significant) = >0.9999, **0.0050, ****<0.0001.

The levels of IgG increased progressively and steadily at all follow-up time points. At D0, the mean antibody titer was 6182.76 ± 1983.23 ng/mL, which rose to a statistically very significant level (*p* = <0.0001) of 6646.62 ± 2089.03 ng/mL on Day 2 (48 hours after the dose), **Figure 1a** On day 30, the mean titer was 8802.67 ± 1569.19 ng/mL; on day 60, 9345.61 ± 1398.10 ng/mL. On day 120, these titers rose to 10193.53 ± 1228.14 ng/mL; on day 180, they rose to 10548.58 ± 1304.90 ng/mL. Increases at all these time points were statistically significant (*p* = <0.0001), **Figure 1a**

Similarly, the mean serum IgA levels increased progressively. At D0, the mean antibody titer was 497.63 ± 277.73 ng/mL, which increased to 489.08 ± 225.70 ng/mL after 48 hours. This increase was not statistically significant (*p* = >0.9999) compared to the increase in IgG levels, **Figure 1b** Also, of lesser statistical significance (p = 0.0050) was the increase on day 30, and the IgA levels were 679.47 ± 278.62 ng/mL, **Figure 1b** After this, the increase in IgA levels was statistically significant (*p* = <0.0001). On day 60, day 120 & day 180, the mean titers were 804.84 ± 332.63 ng/mL, 910.91 ± 349.72 ng/mL & 930.80 ± 333.11 ng/mL, respectively (**Figure 1b**).

### 3.2 Inflammatory cytokines

This study analyzed IL-6 and IFN-γ by ELISA and CRP & hs-CRP by FIA method. These were measured twice in participants, i.e., at D0 and D2. Mean IL-6 at D0 was 92.64 ± 69.10 ng/L which decreased to 82.55 ± 74.90 ng/L at D2. This decrease in IL-6 levels was statistically insignificant (*p* = >0.05), as shown in **Table 1**.

**Table 1:** Pre-vaccination (D0) and post-vaccination (D2) levels of inflammatory cytokines (Statistical test: Mann Whittney).

Inflammatory biomarkers	Reference value	D0 (mean ± SD)	D2 (mean ± SD)	p-value
IL-6 (ng/L)		92.64 ± 69.10	82.55 ± 74.90	>0.05
IFN-γ (ng/mL)		54.70 ± 27.69	46.08 ± 26.15	<0.05
CRP (mg/L)	0-10	6.12 ± 4.42	14.84 ± 27.70	<0.0001
hsCRP (mg/L)	0-1	1.47 ± 1.59	3.52 ± 1.55	<0.0001

The mean IFN-γ at D0 was 54.70 ± 27.69 ng/mL, which decreased to 46.08 ± 26.15 ng/mL at D2. A decrease was observed in IFN-γ, like IL-6, but unlike IL-6, it was statistically significant (*p* = <0.05), though the statistical significance was not as great as seen in **Table 1**.

The mean CRP levels were 6.12 ± 4.42 at D0, which rose to 14.84 ± 27.70 mg/L at D2. Unlike IL-6 & IFN-γ, a statistically significant (*p* = <0.0001) increase was noted in CRP levels (**Table 1**). Similarly, mean hsCRP at D0 was 1.47 ± 1.59 mg/L which increased to 3.52 ± 1.55 mg/L at D2. Similar to CRP, a significant increase (*p* = < 0.0001) was noted in hsCRP levels at D2 (**Table 1**).

### 3.3 Coagulation profile

D-dimers, PT, and APTT were assessed to determine the coagulation profile. D-dimers were assessed using FIA, while PT and APPT were analyzed by manual method.

At D0, the mean D-dimers level was 0.20 ± 0.21 mg/L, which increased to 0.47 ± 0.57 mg/L in at D2. This increase was statistically significant (*p* = <0.005), as shown in **Table 2**.

**Table 2:** Pre-vaccination (D0) and post-vaccination (D2) coagulation profile (Statistical test: Mann Whittney).

Coagulation markers	Reference value	D0 (mean ± SD)	D2 (mean ± SD)	p-value
d-Dimers (mg/L)	<0.50	0.20 ± 0.21	0.47 ± 0.57	<0.005
PT (seconds)	10-13	12.49 ± 2.09	13.51 ± 2.88	<0.05
aPTT (seconds)	25-35	33.02 ± 17.04	34.13 ± 14.11	<0.05

The mean prothrombin time at D0 was 12.49 ± 2.09 seconds, which increased slightly to 13.51 ± 2.88 seconds at D2. The increase was less significant statistically (*p* = <0.05) (**Table 2**). Similarly, the mean APTT at D0 was 33.02 ± 17.04 seconds, which increased to 34.13 ± 14.11 seconds at D2. Like PT, the increases in APTT were also statistically less significant (*p* = <0.05), as shown in **Table 2**.

### 3.4 Cell counts

Cell counts were performed at two time points: D0 corresponds to the time before the vaccine dose, and D2 corresponds to the time 48 hours after the vaccine dose.

The mean lymphocyte count at D0 was 2.34 ± 0.81 *x* 10^3^/μL, which decreased to 1.91 ± 0.53 *x* 10^3^/μL at D2. The decreases in lymphocyte count were statistically significant (p = <0.0005), as shown in **Table 3**. The mean neutrophil count at D0 was 4.33 ± 1.06 *x* 10^3^/μL, which decreased to 4.05 ± 1.04 *x* 10^3^/μL at D2. Unlike lymphocyte count, this decrease in neutrophil counts was not statistically significant (*p* = >0.05) (**Table 3**). The mean platelet count at D0 was 246.25 ± 73.64 *x* 10^3^/μL, which increased to 256.99 ± 76.20 *x* 10^3^/μL at D2. As noted, unlike the lymphocytes and neutrophil counts, an increase was observed in platelet counts, but it was not statistically significant (p = >0.05), as presented in **Table 3**.

**Table 3:** Pre-vaccination (D0) and post-vaccination (D2) cell counts (x 103/μL) (Statistical test: Unpaired t-test).

Cells type	Reference value	D0 (mean ± SD)	D2 (mean ± SD)	p-value
Lymphocytes (10^9^/L)	1.10 - 3.20	2.34 ± 0.81	1.91 ± 0.53	<0.0005
Neutrophil (10^9^/L)	1.80 – 6.30	4.33 ± 1.06	4.05 ± 1.04	>0.05
Platelets (10^9^/L)	150 - 450	246.25 ± 73.64	256.99 ± 76.20	>0.05

## 4 Discussion

This study aimed to assess the effects of the 3rd dose of an mRNA COVID-19 vaccine on antibody response, inflammatory biomarkers, coagulation profile, and blood cell counts. These were the immunoglobulins, i.e., Anti-SARS-CoV-2 IgG and IgA; the inflammation biomarkers, i.e., IL-6, IFN-γ, CRP & hsCRP; the coagulation profile, i.e., D-dimers, PT & APTT and cell counts, i.e., lymphocytes, neutrophils & platelets counts. A total of 68 healthy participants were enrolled who had received two doses of the COVID-19 vaccine earlier, with a second dose six months but no more than eight months than this study.

As the results demonstrate, the anti-SARS-IgG and IgA levels increased after the dose of the mRNA vaccine. Although the increase in IgG levels was rapid, a significant increase was noted even 48 hours after vaccination. Levels of both IgG and IgA didn’t reach a plateau till day 180, i.e., six months after the vaccine dose. This confirms the prolonged protection conferred by the COVID-19 mRNA vaccines. Similar findings are reported by Sabina Zurac et al., who determined the IgG and IgA at day 21 and day 45 and showed that the increase in mean IgA was lower than mean IgG after 2nd dose of the mRNA vaccine at day 21. Another study by Herzberg et al. that investigated the immune response to third dose of an mRNA vaccine also indicated a significant increase in the immune response after 4 weeks of the 3^rd^ dose (Herzberg et al., 2022).

However, in contrast to our study, they showed that the mean IgA levels at day 45 were higher than IgG levels, while in our study, no such increase in levels of IgA compared to IgG was observed throughout the study period (Zurac et al., 2021). As we have no record of the IgG and IgA levels of the participants after the second dose, it can’t be concluded whether the immunoglobulin levels reported before the third dose decreased or not compared to those after the second dose. Some studies in Europe and Japan have shown that the IgG levels had dropped to 10% six months after the second dose (Naaber et al., 2021; Kato et al., 2022). However, a much larger study of more than 18 thousand Polish healthcare workers indicated that mRNA vaccines provided protection till 8–9 months after the second dose (Skorupa et al., 2022). The same study showed that with the 3^rd^ dose, the IgG Levels increased significantly at around the fifth day and maximized on the fourteenth day (Skorupa et al., 2022). In our research, like the study by Monika Skorupa et al (Skorupa et al., 2022), there was an abrupt increase in IgG levels, indicating that the immunity granted by the earlier two doses has significant memory that rapidly boosts upon any future encounter with the virus or any of its components. As shown by the results in **Figure 1a**, a constant rise was observed for six months, and no plateau was reached till that time.

Immune response is always preceded and/or accompanied by inflammation, whether the inflammation is initiated by infection or transfection. Inflammatory markers assessed in our study were IL-6, IFN-γ, CRP & hs-CRP. All these are mutually inclusive, as CRP is an acute inflammatory marker produced by the liver when signaled by IL-6. They both assess low-grade inflammation (Del Giudice and Gangestad, 2018). Also, IFN-γ and IL-6 have been hypothesized to have functionally opposing roles in inflammation, immune response, and cell proliferation (Qi et al., 2013). IFN-γ has pro-inflammatory effects, while those of IL-6 are anti-inflammatory (Qi et al., 2013). The study cited above by Herzberg et al. and another study by Thomas et al. reported that IFN-γ increases significantly after the booster dose suggesting the T-cell immune response. In study by Herzberg et al., they reported that 96.3% of the participants showed detectable T-cell response evident by the rise of IFN-γ. Thomas et al. delved deeper into the matter and reported that significant increase was shown in the IFN-γ along with granzyme B production, neutralizing antibodies and class-switched B cells (Thomas et al., 2025). The stratification protocols and sophisticated techniques of both of these studies gives them increased value and our findings are in accordance with the relative aspects of these studies. In our study, a rise in CRP was noted 48 hours after the third dose of the vaccine, **Table 1**. A survey by Ugur Sahin also reported the same results and found that the CRP levels dropped to normal on day eight after the vaccination (Sahin et al., 2020). hs-CRP also increased with statistical significance (*p* = <0.0001) in our study 48 hours after the vaccine dose. For quite some time, hs-CRP has been considered an independent marker of cardiovascular risks (Moutachakkir et al., 2017). In line with this, several studies have concluded from the published data that there can be a possible association between COVID-19 vaccination and cardiac events, markedly visible in otherwise healthy, younger male populations and with repeated doses of mRNA vaccines (Fatima et al., 2023). Though a temporal association can be established between the vaccine injection and the development of myocarditis, the milder nature of adverse events, low incidence rate, and lack of experimental studies make it difficult to pose a cause-effect association.

COVID-19 infection has been associated with disturbances in coagulation pathways. The disturbed coagulation profile expressed by the PT, APTT, and D-dimers has been a significant indicator of disease severity and is used to guide treatment plans (Mucha et al., 2020). S-protein has been pointed out as the virus’s main antigenic and inflammatory antigen. As the mRNA vaccines contain S-protein or subunits of S-protein, the prolonged antigen presence and excessive immune response can trigger sustained inflammation that can harm the endothelium, thus disturbing anti-thrombogenic properties in multiple vascular beds (Trougakos et al., 2022). Studies have shown remarkable disturbances in coagulation profiles after the vaccine. A narrative review by Emmanuel J. Favaloro demonstrated that in most studies included in the review, D-dimers in patients diagnosed with suspected COVID-19 vaccine-induced thrombotic thrombocytopenia were almost always abnormal (Favaloro, 2021). In our research, like the observations of Favaloro, a statistically significant increase was reported in D-dimer levels (**Table 2**). Our study noted a rise of lesser statistical significance in PT and APTT. The study already mentioned by Favaloro also pointed out that in most cases with COVID-19-induced thrombotic thrombocytopenia, PT and APTT were also deranged (Favaloro, 2021). Another study in Nature by Jiping Liu et al. reported that the mRNA vaccine significantly alters the coagulation profile (Liu et al., 2021). They found that PT was shortened on day 7 after the vaccine, but on days 28 and 42, both PT and APTT were prolonged. They followed the patients and reported that the coagulation profile returned to normal at day 90. These changes in coagulation profile for a significantly longer time suggest that however clinically less significant these changes may look, they should be looked at with great care, especially with repetitive doses and patients at risk for bleeding diathesis.

Another important aspect related to inflammatory, immunologic, and coagulation profiles is the blood cells, as they play an essential role in inflammation, immune response, and the cross-talk between various process stakeholders in the language of cytokines and interleukins and coagulation profile. Our study focused on neutrophils, lymphocytes, and platelet count changes. A decrease of statistical significance in lymphocyte count was noted 48 hours after the vaccine dose (**Table 3**). A study by Ugur Sahin et al. also reported the decrease of a similar pattern in lymphocytes on day two after the vaccine dose that returned to normal on day 8 (Sahin et al., 2020). The same study, on the other hand, had reported an increase in neutrophil count on day 2, returning to normal on day 8 after the vaccine dose (Sahin et al., 2020).

In contrast to the findings of Ugur Sahin et al., a decline in neutrophil count was noted in this study on day 2 after the vaccine dose, but that was statistically not significant (**Table 3**). Our study reported a statistically insignificant increase in platelet count on day 2 after the vaccine dose. Although not statistically significant, the increase in platelet count contrasted with the general observation of a decrease after COVID-19 vaccination. However, rare cases have been reported where patients had presented with thrombocytosis after COVID-19 vaccination, but those vaccines were adenoviral based (Graça et al., 2021).

It is essential to acknowledge the limitations of this study. Firstly, the sample size was small. The small sample size affects the generalizability of these results to wider segments of population with different ethnic, geographic and genetic backgrounds. Secondly, children and old aged patients were not included. In addition, patients with immunodeficiencies, comorbidities, coagulation abnormalities, and inflammatory diseases were not enrolled, and the results cannot be applied to them. Thirdly, the study could not assess the IgG and IgA levels after six months of the vaccine dose. Similarly, changes in inflammatory cytokines and coagulation profile were not followed within 48 hours of the booster dose, and the study cannot shed light on what could have been observed after this specified time.

Further research should be carried out to address these limitations and better understand the kinetics of antibodies, changes in inflammatory markers, coagulation profiles, and cellular components of blood, as well as the interaction of these related mechanisms.

## 5 Conclusions

A booster dose with an mRNA vaccine at the recommended time causes the anti-SARS-CoV-2 IgG and IgA levels to increase rapidly and for at least six months. The increase in inflammatory cytokines and changes in the coagulation profile are consistent with the vaccine’s normal immune induction mechanism. Mild AEFIs were reported in a minority of vaccine recipients. The consistent subclinical changes in inflammatory and coagulation markers suggest that these pathways may be involved in the pathogenesis of severe ARFIs reported with mRNA vaccines.

## Data Availability

The raw data supporting the conclusions of this article will be made available by the authors, without undue reservation.
